# Potential Prognostic Significance of Decreased Serum Levels of TRAIL after Acute Myocardial Infarction

**DOI:** 10.1371/journal.pone.0004442

**Published:** 2009-02-16

**Authors:** Paola Secchiero, Federica Corallini, Claudio Ceconi, Giovanni Parrinello, Stefano Volpato, Roberto Ferrari, Giorgio Zauli

**Affiliations:** 1 Department of Morphology and Embryology, University of Ferrara, Ferrara, Italy; 2 Cardiovascular Institute, Azienda Ospedaliera Universitaria S. Anna, Ferrara, Italy; 3 Medical Statistics Unit, University of Brescia, Brescia, Italy; 4 Department of Experimental and Clinical Medicine, University of Ferrara, Ferrara, Italy; Lerner Research Institute, Cleveland Clinic, United States of America

## Abstract

**Background:**

Since soluble TRAIL exhibits anti-inflammatory and anti-atherosclerotic activities both *in vitro* and in animal models, this study was designed to assess the relationship between the serum levels of TRAIL and clinical outcomes in patients with acute myocardial infarction (AMI).

**Methodology/Principal Findings:**

Levels of TRAIL were measured by ELISA in serial serum samples obtained from 60 patients admitted for AMI, both during hospitalization and in a follow-up of 12 months, as well as in 60 healthy control subjects. Serum levels of TRAIL were significantly decreased in patients with AMI at baseline (within 24 hours from admission), compared with healthy controls, and showed a significant inverse correlation with a series of negative prognostic markers, such as CK, CK-MB and BNP. TRAIL serum levels progressively increased at discharge, but normalized only at 6–12 months after AMI. Of note, low TRAIL levels at the patient discharge were associated with increased incidence of cardiac death and heart failure in the 12-month follow-up, even after adjustment for demographic and clinical risk parameters (hazard ratio [HR] of 0.93 [95% CI, 0.89 to 0.97]; p = 0.001).

**Conclusions/Significance:**

Although the number of patients studied was limited, our findings indicate for the first time that circulating TRAIL might represent an important predictor of cardiovascular events, independent of conventional risk markers.

## Introduction

Although in the serum of patients affected by acute myocardial infarction (AMI) several cytokines, including those belonging to the TNF family members, are known to be elevated [1], only limited information is available on the role of TNF family members in modulating cell death after myocardial ischemia [2]–[4]. In this context, it has been suggested that the CD95 system might be involved in post-ischemic cell death in the heart [2], and it has been reported that the serum levels of TNF-related apoptosis inducing ligand (TRAIL) tend to be reduced in patients with coronary artery disease [5], [6]. Although the cellular source of serum TRAIL has not been clearly established, it is important to point out that TRAIL is expressed as a type-II TNF family transmembrane protein, but its extracellular domain can be released as a soluble cytokine [7]. Interestingly, one study has reported enhanced expression of transmembrane TRAIL on the cell surface of circulating CD3^+^ and CD14^+^ cells after AMI and has documented the expression of two of the four transmembrane receptors for TRAIL, TRAIL-R1 and TRAIL-R2, in human cardiomyocytes [3]. In this respect, however, it should be mentioned that TRAIL-R1 and TRAIL-R2, which contain cytoplasmic “death domains”, not only mediate pro-apoptotic signals but can also promote cell type-dependent pro-survival and proliferation signals [8]–[12]. In particular, with regard to the cardiovascular system, it has been shown that TRAIL can induce apoptotic cell death of vascular endothelial cells only under certain conditions, such as inhibition of the Akt pathway [13]–[20]. Whereas, under standard culture conditions, most of the available studies have clearly demonstrated that recombinant soluble TRAIL rather induces the activation of intracellular signaling pathways, such as ERK/MAPK, Akt and NF-kB, which are known to promote the survival/proliferation of endothelial and vascular smooth muscle cells [8]–[20]. Furthermore, administration of TRAIL significantly counteracted the development and extension of atherosclerotic plaques in an experimental model of atherosclerosis (apoE-null mice) [21].

In light of these experimental data, the aim of the present study was to measure the serum levels of TRAIL in a cohort of AMI patients, both at baseline (within 24 hours from AMI) and in the follow-up (up to 12 months from AMI), and to assess the relationship between TRAIL and short- and long-term incidence of cardiovascular death and/or heart failure (HF). The selection of this composite outcome was based on two independent considerations: i) the same pathophysiological origin of both outcomes (i.e., death of cardiac origin and new-onset HF); ii) analyses by individual outcomes (either death or new-onset HF) was not possible given small sample size, while the composite outcome has allowed increasing the power to detect significant differences in TRAIL levels between groups.

## Methods

### Patients, blood sampling and processing

The study population consisted of 60 AMI patients and 60 healthy control subjects. The AMI patients and the control group did not significantly differ for age, sex and body mass index (BMI), as evaluated by Student's t test (for age and BMI) or chi-square test (for percentage of men). Their demographic, clinical and biochemical profiles are presented in **Table 1**. All participant subjects gave written informed consent. The procedures followed were in accordance with the Declaration of Helsinki and approved by the institutional review board of the University Hospital of Ferrara. Blood samples were collected from all AMI patients at entry (14±9 hours after symptom onset; range, 0.5 to 34 hours), at the day of discharge, and 6 and 12 months after AMI. Blood samples were left in ice for 45 minutes, and then centrifuged at 1700 g at 4°C for 15 minutes. Serum was aliquoted, stored at −80°C and thawed only once before analyses. The patients were admitted to hospital for prolonged (>20 minutes) chest pain accompanied by ST-segment changes of ≥1 mm in ≥1 peripheral leads of the ECG or of ≥2 mm in ≥1 precordial leads. Exclusion criteria were as follows: symptom onset >14 hours before hospital admission, Killip class 4, history of HF, presence of any known ongoing infectious disease, and any known current or past neoplastic or immunological disorder. Occurrence of AMI was confirmed in all patients by a 2-fold upper limit of normal (190 U/L) rise in creatine kinase (CK), with an increased level of CK-MB. None had clinical signs of acute or chronic illness or was receiving any treatment. Patients were followed for 12 months for mortality and morbidity end points. At discharge, medical treatment consisted of nitrate (97%), ACE inhibitors (70%), β-blockers (72%), statins (80%), thienopyridines (74%), diuretics (30%), angiotensin II receptor blockers (18%). Dosages and timing of drug administration were in accordance with current AHA/ACC guidelines. None of the control group had clinical signs of acute or chronic illness or was receiving any treatment.

**Table 1:** Charateristics of the study population.

Variables	Healthy Control Subjects	AMI patients at baseline
	(n = 60)	(n = 60)
Age (yrs)	58.4±10.7	61.1±11.9
Men (%)	78.3	83.3
BMI (kg/m^2^)	25.5±4.5	26.9±3.7
Diabetes (%)	0	22
Killip class >1 (%)	……	33.9
BNP (pg/ml)	……	110.8±86.7
Peak CK-MB (ng/ml)	……	226.9±234.2
Troponin I at peak (ng/ml)	……	113.4±178,3
CRP (mg/dl)	0.8±0.3	3.7±6.8
CK (U/L)	……	2149.4±2022.4

Values given as percentage or mean±SD. BMI = body mass index; BNP = B-type natriuretic peptide; CK = creatine kinase; CK-MB = creatine kinase-MB fraction; CRP = C-reactive protein.

### Biochemical analyses

Serum TRAIL was measured in duplicate by specific, commercially available ELISA kit (R&D Systems, Minneapolis, MN) in accordance with the manufacturer's instructions and analyzed with an ELISA reader at 450 nm. Sensitivity of the assay was 2.86 pg/ml and the intra- and inter-assay coefficients of variation were 3.9% and 6%, respectively. Creatine kinase (CK)-MB fraction in serum was measured on a modular platform (Roche Diagnostics, Mannheim, Germany). The troponin I was measured using an immunofluorescent assay calibrated with spiked serum (Biosite Inc., San Diego, California). C-reactive protein (CRP) was measured by nephelometry from fresh serum according to the method of Behring Diagnostic. B-type natriuretic peptide (BNP) was measured by commercial immunoradiometric assay (Shonoria, Paris, France).

### Follow-up and outcome measures

Patients underwent outpatient visits every 6 months. A minimum follow-up of 12 months was planned. The main outcome of interest was a hierarchical composite of total mortality of cardiac origin and new-onset HF. All deaths were considered to be of cardiac origin (n = 9) unless a noncardiac origin (n = 1) was established clinically or at autopsy. Occurrence of HF, in accordance with previously proposed criteria, required the presence of rest or effort dyspnea and ≥1 of the following: pulmonary rales at lung auscultation, S3 tone, evidence of pulmonary congestion at chest X-ray, new appearance of peripheral edema, or use of diuretics.

### Statistical Analyses

Data are calculated and shown as mean±SD or as median and Interquartile Range (IQR), according to the distribution. Differences in TRAIL mean values across study phases were analyzed using analysis of variance (ANOVA) for repeated measures. Comparisons between control subjects and patients, or between patients with and without adverse clinical outcomes were performed with chi-square test, Student's t test or Wilcoxon test for nonparametric variables. Correlations between TRAIL and variables were estimated using Spearman's correlation coefficient. Survival among groups (above and below median TRAIL values at discharge) was compared by use of the log-rank test, while a Cox proportional-hazard model was used to calculate the crude and adjusted risk estimates for the combined end point at 12 months of follow-up. In the multivariable analyses we adjusted for potential confounders deemed to be clinically relevant, including the following covariates: age, gender, diabetes, Killip class (dichotomous, i.e., Killip class 1 vs. Killip class 2, 3), BMI, CK, CK-MB and BNP. The most “parsimonuous” was selected by mean of the Akaike Information Criterion (AIC), which provides a measure of model quality by simulating the situation where the model is tested on a different data set. The R software (http://www.r-project.org/) was used for the analyses. A two-sided p-value<0.05 has been chosen as statistically significant.

## Results

### TRAIL serum levels are markedly decreased in AMI patients and tend to normalize only 6–12 months after AMI

**Figure 1:**
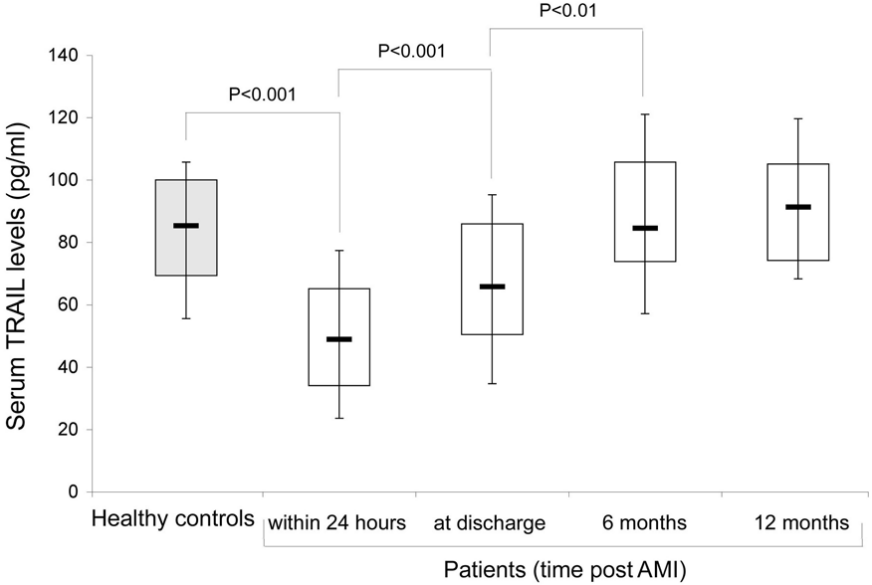
Serum TRAIL levels in AMI patients and healthy individuals. Levels of TRAIL were determined by ELISA in sera from AMI patients (analyzed at the indicated times post AMI) and from healthy control subjects. Horizontal bars are median, upper and lower edges of box are 75th and 25th percentiles, lines extending from box are 10th and 90th percentiles.

The demographic and biochemical characteristics of the study populations analyzed for serum TRAIL levels are illustrated in **Table 1**. At baseline, within 24 hours from admission, the AMI patients showed significantly lower levels of serum TRAIL compared with healthy normal controls, with a median of 47.3 pg/ml (interquartile range 34.1 to 65 pg/ml) (**Figure 1**). In the follow-up analysis of the same patients, we have observed a progressive increase of the TRAIL levels. In particular, at discharge (approximately 5–6 days after admission), the serum levels of TRAIL were already significantly increased, compared to the acute phase, but still significantly (p<0.01) lower with respect to normal controls (**Figure 1**).

After six months post AMI, serum TRAIL levels were further significantly increased, and no significant changes were observed at later time points (12 months; **Figure 1**). Of note, the levels of serum TRAIL measured in the group of patients 6–12 months after AMI were not significantly different from those of healthy control subjects (**Figure 1**).

### TRAIL serum levels show an inverse correlation with biochemical predictors of cardiovascular events

**Figure 2:**
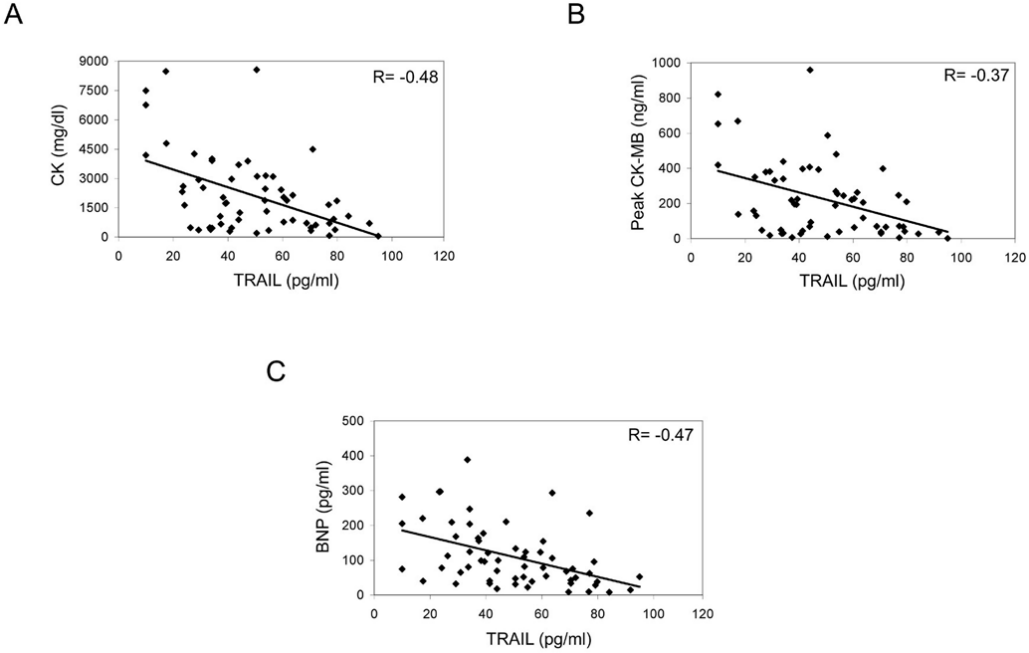
Correlation between serum levels of TRAIL and negative prognostic markers. Inverse correlation between serum levels of TRAIL and CK (A), between TRAIL and CK-MB (B) and between TRAIL and BNP (C) in AMI patients. Coefficients of correlation (R) are indicated.

In the next group of analyses, the serum levels of TRAIL were evaluated with respect to important biochemical markers of cardiovascular related death and HF. We found an inverse correlation between the serum levels of TRAIL, measured within 24 hours after admission, and both CK (R = −0.48; p = 0.002; **Figure 2A**) and CK-MB (R = −0.37; p = 0.007; **Figure 2B**), which represent important markers of degree of myocardial damage immediately after AMI [22]. Moreover, an inverse correlation was also observed between TRAIL and BNP (R = −0.47; p<0.001; **Figure 2C**), a well-known and important prognostic marker of HF [23]. Of note, the association between TRAIL and CK, CK-MB and BNP was significant, as evaluated after multivariate regression adjusted for demographic and clinical parameters (**Tables 2** **–** [Table pone-0004442-t003] **4**). On the other hand, no significant correlations were observed between TRAIL serum levels and CRP, or TRAIL and troponin I (data not shown).

**Table 2:** Association between TRAIL and CK adjusted for demographic and clinical parameters (multivariable linear regression analysis).

Parameters	Estimate	Std. Error	P
TRAIL (pg/ml)	−0.016	0.007	0.019
Age (yrs)	−0.013	0.012	0.249
Gender	0.196	0.356	0.584
BMI (kg/m^2^)	0.047	0.037	0.201
UA	−4.044	0.996	0.000
QWMI	−0.956	0.295	0.002
Diabetes	−0.297	0.330	0.372

BMI = body mass index; QWMI = Q-wave myocardial infarction; UA = unstable angina.

**Table 3 pone-0004442-t003:** Association between TRAIL and CK-MB adjusted for demographic and clinical parameters (multivariable linear regression analysis).

Parameters	Estimate	Std. Error	P
TRAIL (pg/ml)	0.019	0.008	0.031
Age (yrs)	−0.009	0.015	0.521
Gender	0.272	0.448	0.546
BMI (kg/m^2^)	0.040	0.046	0.385
UA	−5.964	1.256	0.000
QWMI	−0.949	0.372	0.014
Diabetes	−0.709	0.416	0.095

BMI = body mass index; QWMI = Q-wave myocardial infarction; UA = unstable angina.

**Table 4:** Association between TRAIL and BNP adjusted for demographic and clinical parameters (multivariable linear regression analysis).

Parameters	Estimate	Std. Error	P
TRAIL (pg/ml)	−0.033	0.008	0.000
Age (yrs)	0.032	0.013	0.013
Gender	0.109	0.388	0.780
BMI (kg/m^2^)	−0.015	0.039	0.707
UA	−0.898	1.083	0.411
QWMI	0.048	0.322	0.883
Diabetes	−0.363	0.353	0.309

BMI = body mass index; QWMI = Q-wave myocardial infarction; UA = unstable angina.

### TRAIL serum levels are significantly lower in AMI patients with primary outcomes with respect to AMI patients without primary outcomes

**Figure 3:**
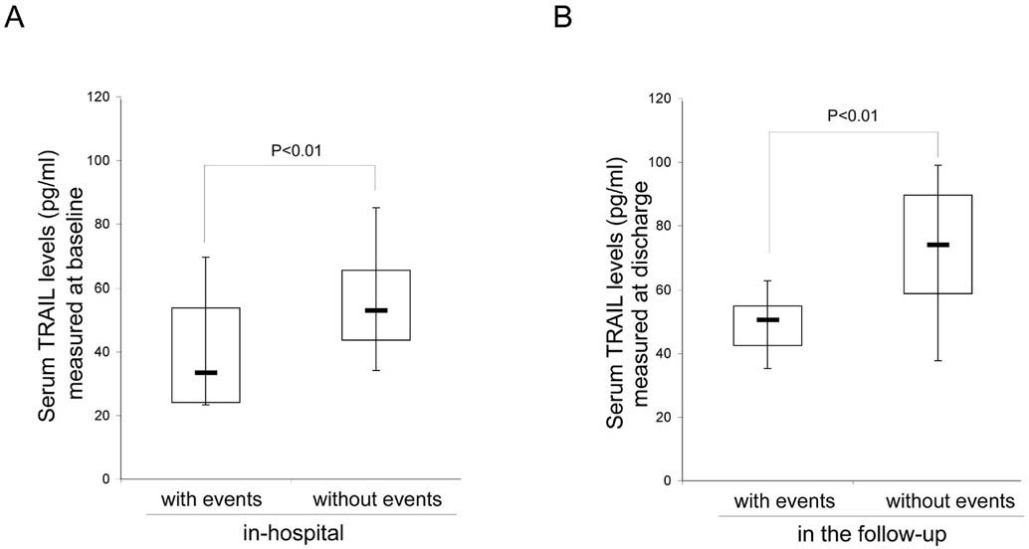
Serum TRAIL levels in AMI patient with respect to adverse clinical events. Serum levels of TRAIL were analyzed in AMI patient who died or experienced HF (with events) either during the acute phase (in-hospital) or in the follow-up (12 months after AMI) with respect to the other AMI patients (without events). Horizontal bars are median, upper and lower edges of box are 75th and 25th percentiles, lines extending from box are 10th and 90th percentiles.

Four patients died during hospitalization as a result of either ventricle free wall rupture or refractory cardiogenic shock, while 7 patients developed new-onset heart failure (HF). It is noteworthy that the TRAIL serum levels evaluated at baseline (within 24 hours) were significantly lower in the subgroup of patients (18.3%) with in-hospital adverse clinical outcomes than in patients without in-hospital cardiovascular events (p = 0.01, **Figure 3A**). The remaining 56 patients were next examined in a follow-up of 12–16 months. During this period, 6 patients died (5 of cardiovascular causes), and 9 patients developed HF. In these 14 out of 56 patients (25%), TRAIL serum levels measured at discharge were profoundly (p<0.01) reduced with respect to the patient group without primary outcomes in the follow-up (**Figure 3B**).

### Decreased TRAIL serum levels represent a negative prognostic factor at 12 months of follow-up in both univariable and multivariable analyses

In next analysis, patients (n = 56) were divided in two groups based on the serum levels of TRAIL at discharge (below or above median values) and the presence of primary outcomes between the two groups in the follow-up was compared by use of the log-rank test. A statistically significant difference (p<0.001) was observed in terms of number of adverse outcomes in the follow-up, suggesting that the low serum levels of TRAIL measured at discharge after AMI represent a determinant of mortality and HF at 12 months of follow-up in this study population. In Cox proportional-hazards regression, after adjustment for potential confounders deemed to be clinically relevant, such as age, gender, BMI, diabetes, Killip>1, CK, CK-MB, and BNP, the TRAIL HR was 0.90 (95% CI, 0.83 to 0.98, p = 0.014). Furthermore, even when Cox proportional-hazards regression analysis was performed after AIC selection, TRAIL still remained an independent and strong predictor of mortality and cardiovascular events after AMI (HR of 0.93, 95% CI, 0.89 to 0.97, p = 0.001) (**Table 5**).

**Table 5:** Cox proportional-hazards regression tests for predictors of death or new-onset HF after AIC selection.

Variables	Analysis for Continuos Variables	
	HR (95% CI)	p value
TRAIL (pg/ml)	0.93 (0.89–0.97)	0.001
Gender	0.14 (0.01–1.44)	0.098
CK	1.001 (1.000–1.001)	0.000

CI = confidence interval; CK = creatine kinase; HR = hazard ratio.

## Discussion

In this pilot study, we have demonstrated that the serum levels of TRAIL were significantly decreased in the early time points after AMI (from admission until discharge) and progressively increased in the follow-up (6–12 months later), reaching levels comparable to those observed in the healthy controls. Our findings confirm and extend previous studies, which showed that the serum levels of TRAIL are decreased in patients affected by coronary artery disease (including angina pectoris and AMI) [5], [6]. While these previous studies suggested that changes in the serum levels of TRAIL might be related to the development of atherosclerotic disease [5], [6], as we have confirmed in a mouse model of atherosclerosis [21], our present study is the first to provide follow-up analysis of the TRAIL levels at various time points after AMI and to indicate that the decreased serum levels of TRAIL might have a negative prognostic significance in AMI patients.

Since both the cellular source of serum TRAIL and the mechanisms of secretion of soluble TRAIL are incompletely understood phenomena [7], [24], it is unclear whether the significant decline in the serum levels of TRAIL reflects a deficit of production or an increased consumption of TRAIL in the acute phase after AMI. With regard to the potential mechanisms of consumption/clearance of TRAIL from the serum, several non-mutually exclusive mechanisms may exist. For instance, it is remarkable that metalloproteases have been reported to be elevated in patients affected by AMI [25] and metalloproteases are able to cleave and inactivate several cytokines/chemokines, such as stromal cell derived factor-1 alpha [26]. Furthermore, it has been previously demonstrated that metalloproteinase inhibitors enhances the biological activity of soluble TRAIL [27]. For these reasons we believe that it would be reasonable and of interest to investigate whether also soluble TRAIL can be cleaved and/or inactivated by metalloproteases.

The major finding of this study is the demonstration that the decreased serum levels of TRAIL, measured within 24 hours after AMI (baseline), were inversely correlated with important prognostic markers for adverse cardiovascular events, such as circulating levels of CK, CK-MB and BNP. However, when adjusting for these and additional variables, deemed to be clinically relevant (i.e., age, gender, BMI, diabetes, Killip>1) in a multivariate analysis, low levels of TRAIL at discharge remained a significant predictor of adverse cardiovascular in the follow-up of 12 months.

Although we are aware that a limitation of this exploratory analysis is the relatively small sample size examined, which does not allow to corroborate the prognostic relevance of TRAIL as a biomarker, several possible mechanisms by which reduced TRAIL serum/plasma levels might contribute to worsen the outcome of AMI can be envisioned. For instance, *in vitro* studies have shown that soluble recombinant TRAIL induces apoptotic cell death of neutrophils [28], [29]. Since neutrophils have been shown to play a key role in worsening the necrosis post-AMI by releasing proteases in the injured tissue [30], [31], it is tempting to speculate that the drastic decline in the serum levels of TRAIL after AMI might induce a temporary impairment in the mechanisms of neutrophil clearance after AMI. In addition, TRAIL exhibits endothelial protective properties, which are related in part to nitric oxide generation by endothelial cells themselves [16] and to an anti-inflammatory activity [19]. An important confirmation that serum TRAIL levels exert anti-inflammatory activity in clinically relevant conditions comes from a recent study in which it has been demonstrated that TRAIL levels progressively increase in the serum of patients after allogenic stem cell transplantation and show a protective effect against graft versus host disease and endothelial cell damage [32].

In conclusion, the results of this pilot study demonstrate for the first time that in AMI patients, in contrast to the raised serum levels of inflammatory cytokines previously documented [1], serum levels of TRAIL are profoundly down-regulated and might carry prognostic information, independently of well-recognized outcome predictors.
